# The anxious addictive narcissist: The relationship between grandiose and vulnerable narcissism, anxiety symptoms and Facebook Addiction

**DOI:** 10.1371/journal.pone.0241632

**Published:** 2020-11-02

**Authors:** Julia Brailovskaia, Elke Rohmann, Hans-Werner Bierhoff, Jürgen Margraf

**Affiliations:** 1 Mental Health Research and Treatment Center, Department of Clinical Psychology and Psychotherapy, Ruhr-Universität Bochum, Bochum, Germany; 2 Department of Social Psychology, Ruhr-Universität Bochum, Bochum, Germany; Chiba Daigaku, JAPAN

## Abstract

Vulnerable narcissism and grandiose narcissism share the core of the narcissistic self but are considered as separate forms of this personality trait. While previous research mainly focused on the mechanisms that connect grandiose narcissism and addictive use of the social platform Facebook, it remained unclear why individuals with enhanced levels of vulnerable narcissism are at risk to develop Facebook Addiction (FA). The present study investigated the links between vulnerable and grandiose narcissism, anxiety symptoms, and FA. In a sample of 327 Facebook users (age: *M*(*SD*) = 23.67(3.96), range: 18–56), both forms of narcissism were positively related to anxiety symptoms and FA. Moreover, the association between both forms of narcissism and FA was partly mediated by anxiety symptoms. Results enter new territory by revealing hidden similarities between vulnerable and grandiose narcissists, emphasizing that similar mechanisms might explain their enhanced risk to develop addictive tendencies of Facebook use. These findings should be considered when assessing individuals at risk for FA and when developing (therapeutic) intervention programs to deal with problematic use of social platforms.

## Introduction

More than two decades ago, Wink described the two faces of narcissism–the grandiose and the vulnerable [1]. Both forms of narcissism share the core of the narcissistic self [2] including the strong belief in the own uniqueness and superiority, a high level of self-love, a strong sense of entitlement, and emphasis on self-serving bias. Attention seeking and the wish for admiration, as well as low agreeableness are common for both grandiose and vulnerable narcissism [3–5]. Younger people have higher levels of both forms of narcissism than older individuals [6–8]. However, there are also considerable differences between grandiose and vulnerable narcissism that emphasize the dual nature of this personality trait [9].

Individuals with enhanced levels of grandiose narcissism typically initiate many superficial social contacts. They present themselves as charming, charismatic, smart, self-confident, open-minded and extraverted interaction partners in social relations [10–12]. They excel in self-promotion. As long as the social contacts remain superficial, narcissistic persons often receive positive feedback from their interaction partners who do not fully recognize their selfishness, their low level of empathy, and their high tendency to manipulate others [11, 12]. This positive feedback confirms their belief of their own grandiosity and fosters their self-esteem further [7, 10, 13–15]. Some previous studies reported grandiose narcissism to be positively linked to subjective happiness, life satisfaction [16, 17], and optimism [18]. In contrast, its relationship to depression symptoms turned out to be negative [19]. Brailovskaia, Bierhoff [20] conducted a series of studies on grandiose narcissism. They found positive (Study 1: r = .32, p < .01) as well as negative (Study 2: r = -.14, p < .05) relationships between this form of narcissism and anxiety symptoms which were assessed with the anxiety subscale of the Depression Anxiety Stress Scales 21 [DASS-21; 21].

Previous researchers termed the vulnerable form of narcissism as maladaptive or pathological [22]. It is positively associated with sensibility, dissatisfaction, and defensiveness [23]. Individuals with enhanced levels of vulnerable narcissism tend to express high self-doubts and anxiety, and to be hypersensitive with respect to social evaluations. They are characterized by insecurity, shyness, and low social competence [2, 9, 22, 24, 25]. Therefore, they typically engage less in social interactions, are unable to promote a positive image of their person and thus perceive less expected attention and admiration from others than individuals with high levels of grandiose narcissism [26–28]. This results in a reduced sense of control and a violation of the fragile self-esteem of vulnerable narcissists [23, 27, 29]. The inability to experience sufficient satisfaction of their high sense of entitlement can foster anxiety symptoms. This closes the vicious circle, because anxiety symptoms contribute to further defensiveness and social withdrawal [1, 19, 24]. Thus, while grandiose narcissists typically achieve public attention and admiration that confirm and foster their belief of own uniqueness and superiority, vulnerable narcissists–due to their low social competence and high anxiety in face-to-face interactions–often suffer from the lack of expected positive feedback and are not able to protect and boost their self-esteem. They experience this as a high burden, because the increase of self-esteem falls under main narcissistic aims [2, 9].

With the emergence of social networking sites (SNSs) people received increased opportunities to engage in various forms of online interaction and self-presentation [30]. In contrast to face-to-face interaction that often requires rapid decisions on own behavior, online activity including self-presentation on SNSs can be precisely planned and controlled [31]. This condition increases the chance to gain positive feedback online from a large audience and thus to enhance the own self-esteem [32]. This corresponds with the narcissistic aims [33] and contributes to the assumption that the use of SNSs could be especially attractive for narcissistic individuals [34–36]. Most studies that investigated the link between narcissism and the use of social online platforms focused on the grandiose form of this personality trait. Considering its high popularity Facebook was the main SNS of choice for such investigations [37, 38]. Individuals with enhanced levels of grandiose narcissism were reported to spend much time on Facebook and to engage in intensive use of this SNS. They have many Facebook friends, frequently upload photos, set “Likes”, write private messages and status updates, and comment the updates of other members of the social platform [6, 39–42]. SNSs constitute the setting in which the social exchange on the basis of mostly superficial online contacts typically provides narcissistic persons with the attention and admiration they are searching for, satisfies their need for popularity, and fosters the experience of positive emotions [16].

Studies that focused on the relationship between vulnerable narcissism and activity on social platforms are comparatively rare. Corresponding to research on grandiose narcissism, available results indicated that persons with enhanced levels of vulnerable narcissism engage in intensive Facebook use by frequently writing private messages, status updates and comments, uploading photos and setting “Likes” [6, 39, 43]. It has been argued that the lack of face-to-face contact on Facebook and the possibility to plan and to control the own self-presentation more comprehensively than in offline interactions might reduce the defensiveness and shyness of vulnerable narcissists in social relations [39]. They tend to use SNSs to regulate negative feelings and for mood improvement [28, 44].

Based on the presented empirical background, it can be concluded that both forms of narcissism are positively linked to social platform use. The more narcissistic individuals engage in active use of SNSs, the more positive feedback including positive comments and “Likes” they receive by their online friends which fosters their self-esteem and increases their feeling of being popular and admired [38]. This seems to be especially of great importance for individuals with enhanced levels of vulnerable narcissism who have less opportunities to get positive feedback in offline interactions because of their defensiveness and social anxiety [9, 39, 44]. In contrast, SNSs are typically only one of many sources of positive feedback for individuals who exhibit increased levels of grandiose narcissism [28].

Previous research that focused on the SNS Facebook described that individuals who consciously engage in intensive use of this platform often experience a high level of flow [45, 46]. Flow is defined as a “state in which people are so involved in an activity that nothing else seems to matter; the experience is so enjoyable that people will continue to do it even at great cost, for the sheer sake of doing it” ([47]; page 4). However, flow experienced during Facebook use was reported to be an antecedent of addictive tendencies [48, 49]. The positive feelings that are linked to the flow experience often contribute to further immersion into the online world, and may cause the development of a close emotional bond to the SNS [50]. This bond is linked to a strong obsessive need to stay permanently online and to use Facebook even though this activity contributes to conflicts in the offline world. This phenomenon was termed Facebook Addiction [FA; 51]. FA is defined by six typical characteristics: salience (permanent thinking about Facebook use), tolerance (more and more time has to be spent on Facebook to experience the same positive emotions as previously with less online time), mood modification (Facebook is typically used for mood improvement without considering alternative ways), relapse (user attempts to reduce the intensity of the own Facebook use; however, the attempt fails and the person returns to old use patterns), withdrawal (experience of unease and nervousness when Facebook may not be used), and conflicts (interpersonal problems in the offline world caused by high intensity of Facebook use) [51, 52].

So far, FA has not been recognized as a formal psychiatric disorder in the Diagnostic and Statistical Manual of Mental Disorders (DSM-5; [53]) and in the International Classification of Diseases (ICD-11; [54]). Due to the relative novelty of this phenomenon, only little longitudinal and experimental research on its etiology is currently available [31, 55, 56]. Moreover, the available studies partly use different instruments to asses FA which limits the comparability of their results [57]. Therefore, similar to other forms of addictive behavior, its potential inclusion in the diagnostic manuals is controversially discussed in the literature [58, 59]. Nevertheless, it should not be ignored that recent longitudinal studies described potential negative consequences of FA for subjective well-being and emphasized the urgent need to investigate the mechanisms that foster its development. FA positively predicted symptoms of depression and insomnia up to six weeks later [60], and it also positively predicted suicide ideation and behavior up to one year later [61].

Based on the currently available findings, the following conclusions can be drawn on the development of FA. The positive association between online flow and FA seems to be particularly strong for individuals who often experience daily stress and immerse into the online world to escape from negative feelings [55, 62]. Especially anxiety symptoms were reported to positively predict the addictive tendencies [57, 63, 64]. Several studies reported a positive relationship between FA and both forms of narcissism. Moreover, it was assumed that narcissistic individuals are at enhanced risk to develop FA [28, 31, 36, 44, 50, 65, 66]. Vulnerable narcissists were described to engage more often in online social interaction and to have higher levels of addictive SNS use than grandiose narcissists [28]. In a recent study, Casale and Fioravanti [44] investigated potential mechanisms that might contribute to the link between narcissism and addictive Facebook use. Results revealed the need for popularity as well as the need to belong to positively mediate the relationship between grandiose narcissism and FA. However, those findings were not replicated for vulnerable narcissism [44]. Therefore, differential factors seem to contribute to the development of addictive use tendencies in individuals with enhanced levels of vulnerable narcissism compared with individuals with increased levels of the grandiose form of narcissism.

Considering the high popularity of SNSs use–particularly the use of Facebook–among narcissistic individuals [32, 38] and the potential negative consequences of FA [60, 61], it seems to be of great importance to understand which mechanisms might connect vulnerable narcissism and addictive Facebook use. This knowledge might, on the one hand, contribute to the explanation of differences between vulnerable und grandiose narcissism. On the other hand, it might support the development of intervention programs that reduce the risk of FA which are specifically tailored to grandiose and vulnerable narcissists. Therefore, the main aim of the current study was to investigate the relationship between narcissism–mainly vulnerable narcissism–and addictive Facebook use.

One of the main characteristics of individuals with increased levels of vulnerable narcissism is anxiety. Inter alia because of their high hypersensitivity and insecurity, they often experience high levels of anxiety in social interactions, and therefore tend to avoid social contacts in the offline world [9, 23]. Nevertheless, due to the narcissistic core that is common for grandiose and vulnerable narcissism, they have a high sense of entitlement, are convinced of their own superiority, and strive for admiration. The inability to satisfy the need for admiration in face-to-face interactions contributes to a further increase of the anxiety symptoms that foster further social withdrawal and defensiveness of vulnerable narcissists [9]. As revealed by previous research, Facebook, which is intensively used by people with high levels of vulnerable narcissism, allows its users to outline a carefully planned and controlled self-presentation [39, 43, 44]. The usage of Facebook as a protected environment facilitates for these individuals the satisfaction of their narcissistic needs. They experience positive feedback that they often miss offline [6, 28]. However, it can be assumed that this positive experience may contribute to further excessive immersion into the online world and to the development of an addictive bond to the SNS that can negatively impact their well-being.

Based on these considerations it might be hypothesized that the more anxiety symptoms vulnerable narcissists experience which are accompanied by higher offline social withdrawal, the more they engage in Facebook activity to compensate the lack of offline contacts. This, however, might enhance their risk to develop FA. Thus, the link between vulnerable narcissism and FA might be mediated by anxiety symptoms. Correspondingly, previous research reported a positive association between anxiety symptoms and FA [57, 63, 64], and between vulnerable narcissism and anxious attachment [9].

Against this background, in the present investigation, we assumed to find a positive relationship between vulnerable narcissism and FA (Hypothesis 1a). Anxiety symptoms were expected to be positively related to vulnerable narcissism (Hypothesis 1b) and to FA (Hypothesis 1c). Moreover, anxiety symptoms were assumed to positively mediate the link between vulnerable narcissism and FA (Hypothesis 2).

Even though the main aim of the current study was to understand the under-researched mechanisms that may contribute to the development of FA in individuals with enhanced levels of vulnerable narcissism, we also included the grandiose form of narcissism in the investigation to be able to reveal potential differences to vulnerable narcissism. Given the previously reported positive association between vulnerable and grandiose narcissism that share the core of the narcissistic self [2], we expected to find a positive relationship between both forms of narcissism (Hypothesis 3a). Additionally, considering earlier findings [50], grandiose narcissism was assumed to be positively related to FA (Hypothesis 3b).

Considering previous inconclusive results about the relationship between grandiose narcissism and anxiety symptoms [20], two exploratory research questions were formulated:

Is grandiose narcissism related to anxiety symptoms? (Research Question 1)

Do anxiety symptoms mediate the relationship between grandiose narcissism and FA? (Research Question 2)

## Materials and methods

### Procedure and participants

The current sample included 327 Facebook users from Germany (72.8% women; age (years): M(SD) = 23.57 (3.96), range: 18–56; occupation: 81.3% university students, 18.7% employed; marital status: 48.9% single, 48.6% with romantic partner, 2.4% married). Participants were recruited by participation invitations displayed at several German universities and at public places, like bakeries. The requirement for participation, which was voluntary and not compensated, was a current Facebook membership. The Ethics Committee of the faculty of psychology of the Ruhr-Universität Bochum approved the implementation of the present study (approval number: 460). The present study was conducted in according to the principles expressed in the Declaration of Helsinki. All participants were fully informed about the study and provided informed consent to participate online. A priori conducted power analyses (G*Power program, version 3.1) indicated that a total sample size of N = 92 was sufficient for valid results (power > .80, α = .05, medium effect size: f^2^ = .15; cf., [67]). The dataset used in the present study is available in S1 Dataset.

### Measures

#### Vulnerable narcissism

The abridged version of the Narcissistic Inventory (NI-R-36; [9]) assessed vulnerable narcissism. The 36 items (e.g., “Other people would be really amazed if they knew about my talents”) are rated on a 5-point Likert scale (1 = not at all true, 5 = completely true; current reliability: Cronbach’s α = .94). The higher the mean score of the items, the higher the level of vulnerable narcissism.

#### Grandiose narcissism

To assess the level of grandiose narcissism the brief version of the Narcissistic Personality Inventory (G-NPI-13; [20]) was included. This instrument consists of 13 items that are rated in forced-choice format (0 = non-narcissistic: e.g., “I am not particularly interested in looking at myself in the mirror”, 1 = narcissistic: e.g., “I like to look at myself in the mirror”; current reliability: Kuder-Richardson (KR-20) = .68). Higher sum scores indicate higher levels of grandiose narcissism.

#### Anxiety symptoms

The anxiety subscale of the Depression Anxiety Stress Scales 21 (DASS-21; [21]) measured anxiety symptoms over the previous week with seven items (“I felt scared without any good reason”) that are rated on a 4-point Likert scale (0 = did not apply to me at all, 3 = applies to me very much or most of the time; current reliability: Cronbach’s α = .89). The higher the sum score, the higher the level of anxiety symptoms.

#### Facebook Addiction (FA)

The brief version of the Bergen Facebook Addiction Scale (BFAS; [51]) assessed the level of FA over the time frame of the past year. This measure includes six items (e.g., “Felt an urge to use Facebook more and more?”) according to the six core addiction features (i.e., salience, tolerance, mood modification, relapse, withdrawal, conflict). Items are rated on a 5-point Likert scale (1 = very rarely, 5 = very often; current reliability: Cronbach’s α = .93). Higher sum scores indicate higher levels of FA.

### Statistical analyses

Statistical analyses were conducted with the Statistical Package for the Social Sciences (SPSS 24) and the macro Process version 2.16.1 (www.processmacro.org/index.html; [68]). First, descriptive statistics of the investigated variables and zero-order bivariate correlations were computed. Next, to assess the predictive effect of both forms of narcissism and anxiety symptoms on FA, a three-step hierarchical regression analysis (CI 95%) was calculated. FA served as the outcome variable of the model. Age and gender were included as control variables in Step 1; in Step 2, vulnerable narcissism and grandiose narcissism were added; anxiety symptoms were added in Step 3. The model was not threatened by multicollinearity (all values of tolerance > .25, all variance inflation factor values < 5 [69]). Then, two mediation analyses (model 4) were calculated. Both mediation models included FA as outcome and anxiety symptoms as mediator. While in the first model, vulnerable narcissism was considered as predictor, grandiose narcissism was included as predictor in the second model. The covariates age and gender were controlled for in both models. Additionally, grandiose narcissism was controlled for in the first model, and vulnerable narcissism was controlled for in the second model. This allowed the investigation of the specific relationship between vulnerable narcissism, anxiety symptoms and FA, while controlling for grandiose narcissism, and vice versa [68]. Path c (the total effect) denoted the basic relationship between vulnerable (grandiose) narcissism and FA. The relationship between vulnerable (grandiose) narcissism and anxiety symptoms was denoted by path a; the link between anxiety symptoms and FA was denoted by path b. The indirect effect was represented by the combined effect of path a and path b. The association between vulnerable (grandiose) narcissism and FA after the inclusion of anxiety symptoms in the model was denoted by path c’ (the direct effect). The magnitude of the mediation effect was assessed by the bootstrapping procedure (10.000 samples) that provides bias corrected bootstrap confidence intervals (CI 95%). P_M_ (the ratio of indirect effect to total effect) served as the mediation effect measure.

## Results

Table 1 summarizes the descriptive statistics and correlations of the investigated variables. The correlational results indicate that vulnerable narcissism, grandiose narcissism, anxiety symptoms and FA were significantly positively associated with each other.

**Table 1:** Descriptive statistics and correlations of vulnerable and grandiose narcissism, anxiety symptoms and Facebook Addiction.

	M (SD)	Min–Max	(2)	(3)	(4)
(1) Vulnerable Narcissism	2.67 (.66)	1.00–4.72	.57**	.57**	.56**
(2) Grandiose Narcissism	4.42 (2.68)	0–13		.41**	.46**
(3) Anxiety Symptoms	2.72 (4.02)	0–20			.73**
(4) Facebook Addiction	8.69 (4.69)	6–29			

N = 327; M = Mean; SD = Standard Deviation; Min = Minimum; Max = Maximum

**p < .01.

Results of the regression analysis are summarized in Table 2. The overall model explained 57.3% of the variance. Both forms of narcissism and anxiety symptoms served as significant predictors of FA. Anxiety symptoms had the strongest predictive effect. The effect of vulnerable narcissism was remarkably stronger than the effect of grandiose narcissism (see Table 2).

**Table 2:** Hierarchical regression analysis with Facebook Addiction as outcome, vulnerable narcissism, grandiose narcissism and anxiety symptoms as predictors; controlling for age and gender.

	ß	95% CI	t	Adjusted R^2^	Changes in R^2^
Step 1, F(2,324) = 9.002, p < .001				.05	.05
Age	.12*	[.02, .27]	2.25		
Gender	.17**	[.68, 2.96]	3.15		
Step 2, F(4,322) = 44.942, p < .001				.35	.31
Vulnerable Narcissism	.43**	[2.30, 3.83]	7.90		
Grandiose Narcissism	.19**	[.15, .53]	3.50		
Step 3, F(5,321) = 88.501, p < .001				.57	.22
Anxiety Symptoms	.58**	[.57, .78]	13.00		

N = 327; ß = standardized coefficient beta; CI = confidence interval

**p < .01

*p < .05.

**Fig 1:**
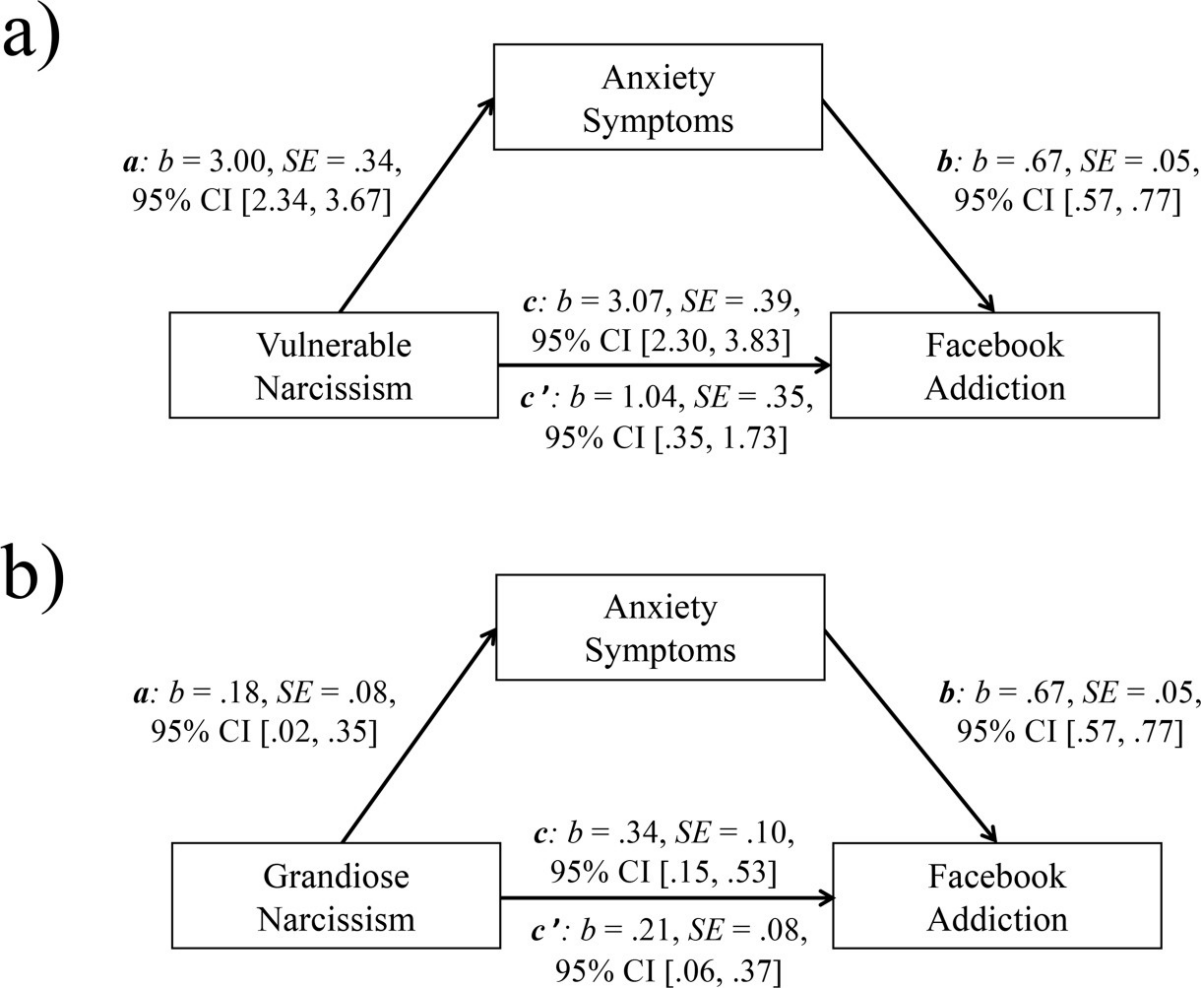
a. Mediation model including vulnerable narcissism (predictor), anxiety symptoms (mediator), and Facebook Addiction (outcome). b. Mediation model including grandiose narcissism (predictor), anxiety symptoms (mediator), and Facebook Addiction (outcome). (c = total effect, c’ = direct effect; b = standardized regression coefficient, SE = standard error, CI = confidence interval).

Fig 1 presents the results of both bootstrapped mediation analyses. The results shown in Fig 1A indicate that anxiety symptoms partly mediated the positive relationship between vulnerable narcissism and FA (total effect, c: p < .0001; direct effect, c’: p = .0033). The indirect effect (ab) was significant (b = 2.03, SE = .40, 95% CI [1.28, 2.87]; P_M_: b = .66, SE = .10, 95% CI [.47, .88]). As shown in Fig 1B, anxiety symptoms also partly mediated the positive relationship between grandiose narcissism and FA (total effect, c: p = .0005; direct effect, c’: p = .0071). The indirect effect (ab) was significant (b = .12, SE = .07, 95% CI [.01, .27]; P_M_: b = .37, SE = .223, 95%CI [.01, .72]). Thus, similar result patterns were found for both forms of narcissism. However, the effect found for vulnerable narcissism was remarkably stronger than the effect found for grandiose narcissism.

## Discussion

Narcissistic individuals often tend to intensively use the SNS Facebook that enables them to experience admiration as special persons. However, this positive feedback may contribute to the development of addictive tendencies that may impact their well-being negatively [60, 65, 70]. The significant findings of the current study confirm our hypotheses and contribute to a better understanding of the mechanisms that connect both forms of narcissism with FA.

In line with previous findings [36, 44], vulnerable narcissism was positively associated with FA (confirmation of Hypothesis 1a). Individuals with a high level of this form of narcissism often engage in intensive Facebook activity [6, 43]. This can be explained as following. Because of their enhanced defensiveness and low social competence vulnerable narcissists are typically not able to promote a positive image of their person in face-to-face social interactions. Consequently, their strong need for attention and admiration remains unsatisfied [23, 29]. On Facebook, they have enough time to create and control their social interactions and self-presentation. This condition increases the probability to receive positive feedback from other users and thus to compensate its lack in the offline world [6, 28, 44].

Furthermore, in accordance with our expectations, vulnerable narcissism was positively related to anxiety symptoms (confirmation of Hypothesis 1b). In contrast to grandiose narcissists who often present themselves as extraverted interaction partners, vulnerable narcissists are characterized by shyness and insecurity [23]. They have a fragile self-esteem and show enhanced anxiety symptoms especially in social situations where they cannot control their self-presentation [27, 71]. Thus, it is not surprising that current results reveal that the higher the level of this form of narcissism which is also termed as the maladaptive narcissism [22], the higher the level of anxiety symptoms.

Anxiety symptoms were also positively related to FA (confirmation of Hypothesis 1c). Previous research that focused on problematic use of SNSs such as Facebook [50, 63, 72], and on problematic smartphone use [73–75] described individuals with increased levels of anxiety symptoms who are often overwhelmed by their offline obligations to consider the use of social media as a possibility to at least temporally escape their daily stress and problems. The social interaction with online friends enables them to experience relief and positive emotions that they often miss offline [76]. However, in the longer term this seems to be a maladaptive coping strategy that, on the one hand, may foster the development of addictive symptoms. On the other hand, it may foster offline interpersonal conflicts because the intensive media use interferes with attending to their obligations at home and at work. In addition, because of the shift of interest to the online world interest to the offline world might be reduced leading to impairments of social skills and increased social insecurity offline. As a consequence, these maladaptive coping strategies further enhance the need to escape into the online world [50, 74].

As hypothesized, anxiety symptoms positively mediated the relationship between vulnerable narcissism and FA (confirmation of Hypothesis 2). This finding might be interpreted as follows. Due to their low social competence, vulnerable narcissists often do not receive the attention and admiration they are searching for to satisfy their high sense of entitlement. This negative experience contributes to their enhanced levels of (social) anxiety [77]. Increased anxiety can further negatively impact their offline self-presentation and social interactions, and is likely to further reduce their probability to gain positive feedback which is of particular importance for the self-regulation of narcissistic individuals [1]. Therefore, vulnerable narcissists seem to be in a vicious circle without a possibility to protect their fragile self-esteem as long as they engage in offline social interactions.

The advent of social platforms provided individuals in general with the possibility to engage in social interaction without face-to-face contact. This offer proves to be particularly valuable for people who exhibit increased levels of vulnerable narcissism. Similar to grandiose narcissists, they search for attention and admiration to confirm and to foster their belief of own uniqueness and superiority [4, 5]. Their lack in offline interactions increases their anxiety symptoms [15]. This contributes to a turn to online interactions where the chance to get positive feedback from a large audience is significantly higher. In line with these considerations, previous research reported a close positive link between vulnerable narcissism and use of Facebook [6, 39, 43, 44]. On the social platform, vulnerable narcissists can exactly plan and control their self-image [39]. This might reduce their anxiety to be negatively evaluated by others because of the improved control over the social situation that is enabled online. And they might receive the attention and admiration that they are missing offline. However, considering the present findings, the high use intensity and the positive emotions experienced during the online usage may contribute to the development of an addictive bond to the social platform that may negatively impact well-being [60]. Thus, considering the current significant mediation model, it may be assumed that the higher the level of vulnerable narcissism, the higher the anxiety level and the higher the risk to develop tendencies of addictive Facebook use. Note that while previous research reported the need for popularity and the need to belong to mediate the relationship between grandiose narcissism and FA, these findings were not replicated for vulnerable narcissism [44]. It remained an open question which mechanisms underlie the positive association between vulnerable narcissism and FA. Therefore, present findings significantly extend available knowledge about these mechanisms by pointing to the mediating role of anxiety symptoms.

Based on previously reported results which indicate that online behavior of vulnerable narcissists resemble the online behavior of grandiose narcissists [6, 39], the grandiose form of narcissism was additionally included in the present investigation. Results revealed a positive relationship between both forms of this personality trait (confirmation of Hypothesis 3a). Despite their specific characteristics, the vulnerable and the grandiose narcissism share the core of the narcissistic self which may explain this positive association [9].

Moreover, in correspondence with previous results [65, 66], grandiose narcissism was positively related to FA (confirmation of Hypothesis 3b). Note that earlier research explained the enhanced risk of grandiose narcissists to develop addictive tendencies of Internet use in general [35], of online gaming [78], and specifically of Facebook use inter alia by the satisfaction of their need for popularity that fosters further excessive online activity and the development of an emotional bond to the source of the positive experiences [44]. Research that considered the valence of the relationship between grandiose narcissism and anxiety symptoms revealed inconclusive results [20]. In the current study, which employed the anxiety subscale of the DASS-21 [21], grandiose narcissism was positively related to anxiety symptoms (see Research Question 1). Moreover, anxiety symptoms positively mediated the association between grandiose narcissism and FA (see Research Question 2). Thus, while mechanisms that contribute to the relationship between grandiose narcissism and FA (i.e., need for popularity, need to belong) do not explain the link between vulnerable narcissism and FA [44], mechanisms that contribute to the association between the vulnerable form of narcissism and FA are also applicable to grandiose narcissism. However, as shown by the results of the regression analysis and the mediation analyses, vulnerable narcissism seems to contribute to the addictive tendencies to a greater extent than grandiose narcissism. This finding emphasizes the rather pathological character of vulnerable narcissism [79].

The present findings are of great interest from a theoretical and applied point of view. They contribute to a better understanding of the two forms of the personality trait narcissism, their relationship with each other and with problematic online activity. The results allow the assumption that the use of SNSs can in the short-term support individuals with enhanced levels of vulnerable narcissism to escape the vicious circle of missing satisfaction of their sense of entitlement, occurrence of anxiety symptoms and social withdrawal in the offline world. The immersion into the online world contributes to the fulfillment of their narcissistic needs. However, in the longer-term, this maladaptive coping strategy can foster addictive tendencies and therefore negatively impact their well-being and everyday life. Furthermore, the present findings reveal that even though–in contrast to vulnerable narcissists–grandiose narcissists are characterized by a self-confident and extraverted self-presentation offline and online, there seem to be hidden characteristics of insecurity and anxiety associated with this form of narcissism that enhance the risk to develop addictive tendencies on Facebook. These risk factors are typical attributes of vulnerable narcissists, but their occurrence is rather remarkable for the grandiose form of this personality trait. Therefore, it may be speculated that grandiose narcissism and vulnerable narcissism share more similarities than previously assumed. However, in contrast to vulnerable narcissists, grandiose narcissists are better able to control and mask their anxiety in the offline world.

Facebook offers its users many different ways of social interaction and self-presentation in front of a large audience. Typically, grandiose narcissists use these possibilities successfully and receive a lot of positive feedback [80]. To maintain the high level of this feedback, they have to permanently control their usage steps. The huge Facebook audience shouldn’t recognize their low agreeableness and their tendency to manipulate others. Previous research reported that the many opportunities of interaction on Facebook may overload users and foster the experience of stress symptoms [81]. Therefore, it might be that in the longer-term individuals with enhanced levels of grandiose narcissism feel overwhelmed by the opportunities and requirements of Facebook use which impede their efforts to maintain the control over their anxiety symptoms and to suppress them adequately. As a consequence, the anxiety symptoms may become obvious and foster the development of addictive tendencies. This assumption should be further considered in longitudinal studies to investigate the hypothesized causality.

Thus, it can be concluded that, independent from the form of narcissism, individuals who exhibit an increased level of this personality trait are at risk to develop FA. Similar mechanisms might apply to both vulnerable and grandiose narcissists to explain this process of mediation: The higher the narcissism level, the more anxiety symptoms are experienced that foster the development of addictive tendencies. However, this effect seems to be stronger for the vulnerable form of narcissism. These findings might be applied in clinical screenings. They show that not only vulnerable narcissists, but also persons with high levels of grandiose narcissism may be at risk to develop enhanced anxiety symptoms. Grandiose narcissists might be able to hide their anxiety over a longer period of time and therefore the reasons for their suffering might remain unrecognized. The screening could disclose their hidden anxiety and contribute to the provision of an adequate therapeutic support.

Moreover, the current results shed at least partly light on the mechanisms that contribute to the development of FA in the narcissistic user group. This knowledge might be useful for developing intervention programs to prevent addictive media use and to protect the well-being of narcissistic individuals. A successful handling of the anxiety symptoms might reduce the risk of developing an emotional bond to the SNSs in individuals with enhanced levels of both vulnerable and grandiose narcissism.

However, note that in both mediation models only a partial mediation effect was obtained. Thus, it can be assumed that anxiety symptoms are an important, but not the sole factor that explains the enhanced risk of narcissistic individuals to develop FA. Further factors can additionally contribute to this relationship. Their identification and successful handling could decrease the addictive tendencies and protect the well-being of narcissistic people. Therefore, it seems reasonable to include further potential mediators–for example depression symptoms–in the endeavor to investigate the relationship between narcissism and FA. Considering previous findings that showed the need for popularity as well as the need to belong to mediate the relationship between grandiose narcissism and FA [44], it seems to be reasonable to replicate this previous investigation by additionally including anxiety symptoms in the mediation model.

### Limitations and further research

The current study provides a deeper understanding of the mechanisms involved in the association between vulnerable and grandiose narcissism, anxiety symptoms, and addictive Facebook use. However, there are some limitations that are important to mention when interpreting the present results.

First, given the cross-sectional nature of the present data, only hypothetical conclusions about the causality of the described relationships should be drawn [82]. Future research is recommended to replicate our findings with a longitudinal (i.e., several measurement time points over a longer period) and experimental design to be able to draw truly causal conclusions about mechanisms that may influence the vulnerability of narcissistic individuals to develop addictive tendencies of Facebook use. For example, anxiety symptoms might be reduced by the implementation of a (therapeutic) intervention. If the reduction of the anxiety symptoms contributes to the decrease of FA especially in individuals with enhanced levels of narcissism, then strong conclusions about causal impact can be drawn.

Second, the comparably young and mostly female composition of the sample limits the generalizability of the current findings. To partly tackle this limitation age and gender were controlled for in the analyses. Nevertheless, future researchers are suggested to replicate present findings on the basis of a more balanced gender and age composition of the sample. Note that earlier research reported male individuals to have higher levels of grandiose narcissism than female individuals [15, 83]. However, recent research found no gender differences assuming a convergence of the narcissism level due to the convergence of gender roles in the modern society [84]. Findings considering vulnerable narcissism are inconsistent. While some studies described no gender differences [2, 29, 85], other reported female individuals to have higher levels of this form of narcissism than male individuals [9, 84].

Third, grandiose narcissism was assessed with the internationally well-established and previously validated brief form of the Narcissistic Personality Inventory [20, 86]. However, in the present investigation this measure reached only a satisfactory reliability that might impact current findings. This corresponds to previous research [87] that investigated the psychometric properties of the original 40-item version of the Narcissistic Personality Inventory [88]. The authors [87] highlighted the potential inadequacy of this measure for assessing two of its subscales (i.e., “Entitlement” and “Superiority”) and suggested the usage of other more reliable instruments such as the Narcissistic Admiration and Rivalry Questionnaire [89]. If for instance, the trait entitlement which can be considered as a component of narcissism [88] or as a stand-alone psychological construct [90] is of specific interest, the use of the corresponding eight items of the Equity Performance Questionnaire [91] is recommended [92, 93]. In addition, in the current study as in the previous research by Brailovskaia, Bierhoff [20] the anxiety subscale of the DASS-21 [21] was employed that represents a well-established anxiety questionnaire. Nevertheless, the employment of alternative measures of anxiety would help to validate the generalizability of the present results across several measures of anxiety.

Fourth, previous research on problematic or addictive social platform use mostly focused on the SNS Facebook [57]. Therefore, mechanisms that associate narcissism and addictive Facebook use were investigated in the present work. However, recent studies reported also use of other SNSs such as Instagram to be linked to addictive tendencies that might negatively impact subjective well-being [70, 94]. Therefore, it should be investigated whether present findings about the mediation effect of anxiety symptoms may generalize to other social platforms, or whether there are unique for Facebook. Additionally, even though terms such as Facebook Addiction or Social Media Addiction are commonly used to describe the problematic emotional bond to the online world [38, 51, 70, 95], it is important to consider that this phenomenon has currently not been recognized as a formal psychiatric disorder in the DSM-5 [53] and the ICD-11 [54]. Thus, conclusions about the mental health state of the investigated sample should be considered with caution.

## Conclusion

Current results indicate that vulnerable as well as grandiose narcissists may be at risk to develop tendencies of addictive Facebook use. Anxiety symptoms may positively mediate this association for both forms of narcissism. Thus, it may be inferred that the intensive use of Facebook typically reported for narcissistic individuals is at least partly driven by anxiety that may foster the development of addictive tendencies. Longitudinal research should investigate these preliminary conclusions.

## Supporting information

S1 Dataset Dataset used for analyses in present study. (SAV)

## References

[1] Wink P. Two faces of narcissism. Journal of personality and social psychology. 1991;61(4): 590–7. doi: 10.1037//0022-3514.61.4.590 1960651

[2] Rohmann E, Brailovskaia J, Bierhoff H-W. The framework of self-esteem: Narcissistic subtypes, positive/negative agency, and self-evaluation. Current Psychology. 2019: 1–8. doi: 10.1007/s12144-019-00431-6

[3] Campbell WK, Rudich EA, Sedikides C. Narcissism, self-esteem, and the positivity of self-views: Two portraits of self-love. Personality and Social Psychology Bulletin. 2002;28(3): 358–68. doi: 10.1177/0146167202286007

[4] Campbell WK, Brunell AB, Finkel EJ. Narcissism, interpersonal self-regulation, and romantic relationships: An agency model approach. In: Vohs KD, Finkel EJ, editors. Self and relationships: Connecting intrapersonal and inter-personal processes. New York: Guilford; 2006. p. 57–83

[5] Emmons RA. Narcissism: theory and measurement. Journal of Personality and Social psychology. 1987;52(1): 11–7. doi: 10.1037//0022-3514.52.1.11 3820065

[6] Brailovskaia J, Bierhoff H-W. The Narcissistic Millennial Generation: A Study of Personality Traits and Online Behavior on Facebook. Journal of Adult Development. 2020;27(1): 23–35. doi: 10.1007/s10804-018-9321-1

[7] Twenge JM, Konrath S, Foster JD, Campbell WK, Bushman BJ. Egos inflating over time: A cross‐temporal meta‐analysis of the Narcissistic Personality Inventory. Journal of Personality. 2008;76(4): 875–901. doi: 10.1111/j.1467-6494.2008.00507.x 18507710

[8] Foster JD, Campbell WK, Twenge JM. Individual differences in narcissism: Inflated self-views across the lifespan and around the world. Journal of Research in Personality. 2003;37(6): 469–86. doi: 10.1016/S0092-6566(03)00026-6

[9] Rohmann E, Neumann E, Herner MJ, Bierhoff H-W. Grandiose and vulnerable narcissism. European Psychologist. 2012;17: 279–90. doi: 10.1027/1016-9040/a000100

[10] Paulhus DL. Normal narcissism: Two minimalist accounts. Psychological Inquiry. 2001;12(4): 228–30.

[11] Watson P, Grisham SO, Trotter MV, Biderman MD. Narcissism and empathy: Validity evidence for the Narcissistic Personality Inventory. Journal of Personality Assessment. 1984;48(3): 301–5. doi: 10.1207/s15327752jpa4803_12 16367529

[12] Foster JD, Campbell WK. Are there such things as “narcissists” in social psychology? A taxometric analysis of the Narcissistic Personality Inventory. Personality and Individual Differences. 2007;43(6): 1321–32. doi: 10.1016/j.paid.2007.04.003

[13] Rudich EA, Sedikides C, Campbell WK. Narcissism, Self-Esteem, and the Positivity of Self-views: Two Portraits of Self-Love. Journal of the Acoustical Society of America. 2002;73: 1354–60.

[14] Kernis MH, Sun C-R. Narcissism and reactions to interpersonal feedback. Journal of Research in Personality. 1994;28(1): 4–13. doi: 10.1006/jrpe.1994.1002

[15] Morf CC, Rhodewalt F. Unraveling the paradoxes of narcissism: A dynamic self-regulatory processing model. Psychological Inquiry. 2001;12(4): 177–96. doi: 10.1207/S15327965PLI1204_1

[16] Brailovskaia J, Margraf J. I present myself and have a lot of Facebook-friends–Am I a happy narcissist!? Personality and Individual Differences. 2019;148: 11–6. doi: 10.1016/j.paid.2019.05.022

[17] Brailovskaia J, Margraf J. Comparing Facebook users and Facebook non-users: relationship between personality traits and mental health variables–an exploratory study. PloS ONE. 2016;11(12): e0166999. doi: 10.1371/journal.pone.0166999 27907020 PMC5131958

[18] Hickman SE, Watson PJ, Morris RJ. Optimism, pessimism, and the complexity of narcissism. Personality and Individual Differences. 1996;20(4): 521–5. doi: 10.1016/0191-8869(95)00223-5

[19] Rathvon N, Holmstrom RW. An MMPI-2 portrait of narcissism. Journal of Personality Assessment. 1996;66(1): 1–19. doi: 10.1207/s15327752jpa6601_1 8576824

[20] Brailovskaia J, Bierhoff H-W, Margraf J. How to identify narcissism with 13 items? Validation of the German Narcissistic Personality Inventory-13 (G-NPI-13). Assessment. 2017: 1–15. doi: 10.1177/1073191117740625 29117708

[21] Lovibond PF, Lovibond SH. The structure of negative emotional states: comparison of the Depression Anxiety Stress Scales (DASS) with the Beck Depression and Anxiety Inventories. Behaviour Research and Therapy. 1995;33(3): 335–43. doi: 10.1016/0005-7967(94)00075-u . 7726811

[22] Miller JD, Campbell WK. Comparing clinical and social‐personality conceptualizations of narcissism. Journal of Personality. 2008;76(3): 449–76. doi: 10.1111/j.1467-6494.2008.00492.x 18399956

[23] Dickinson KA, Pincus AL. Interpersonal analysis of grandiose and vulnerable narcissism. Journal of Personality Disorders. 2003;17(3): 188–207. doi: 10.1521/pedi.17.3.188.22146 12839099

[24] Fossati A, Somma A, Borroni S, Pincus AL, Markon KE, Krueger RF. Profiling Pathological Narcissism According to DSM–5 Domains and Traits: A Study on Consecutively Admitted Italian Psychotherapy Patients. Psychological Assessment. 2016;29(11): 1400–11. doi: 10.1037/pas0000348 27336839

[25] Krizan Z, Herlache AD. The narcissism spectrum model: A synthetic view of narcissistic personality. Personality and Social Psychology Review. 2018;22(1): 3–31. doi: 10.1177/1088868316685018 28132598

[26] Cain NM, Pincus AL, Ansell EB. Narcissism at the crossroads: Phenotypic description of pathological narcissism across clinical theory, social/personality psychology, and psychiatric diagnosis. Clinical Psychology Review. 2008;28(4): 638–56. doi: 10.1016/j.cpr.2007.09.006 18029072

[27] Pincus AL, Roche MJ. Narcissistic grandiosity and narcissistic vulnerability. In: Campbell WK, Miller JD, editors. Handbook of narcissism and narcissistic personality disorder: Theoretical approaches, empirical findings, and treatments New York, NY: Wiley & Sons; 2011. p. 31–40

[28] Casale S, Fioravanti G, Rugai L. Grandiose and vulnerable narcissists: who is at higher risk for social networking addiction? Cyberpsychology, Behavior, and Social Networking. 2016;19(8): 510–5. doi: 10.1089/cyber.2016.0189 27362922

[29] Rohmann E, Hanke S, Bierhoff H-W. Grandiose and Vulnerable Narcissism in Relation to Life Satisfaction, Self-Esteem, and Self-Construal. Journal of Individual Differences. 2019. doi: 10.1027/1614-0001/a000292

[30] Boyd DM, Ellison NB. Social network sites: Definition, history, and scholarship. Journal of Computer-Mediated Communication. 2007;13(1): 210–30. doi: 10.1111/j.1083-6101.2007.00393.x

[31] Brailovskaia J, Margraf J. Facebook Addiction Disorder (FAD) among German students–a longitudinal approach. PLoS ONE. 2017;12(12): e0189719. doi: 10.1371/journal.pone.0189719 29240823 PMC5730190

[32] Gentile B, Twenge JM, Freeman EC, Campbell WK. The effect of social networking websites on positive self-views: An experimental investigation. Computers in Human Behavior. 2012;28(5): 1929–33. doi: 10.1016/j.chb.2012.05.012

[33] Sedikides C, Rudich EA, Gregg AP, Kumashiro M, Rusbult C. Are normal narcissists psychologically healthy?: self-esteem matters. Journal of Personality and Social Psychology. 2004;87(3): 400–16. doi: 10.1037/0022-3514.87.3.400 15382988

[34] Mehdizadeh S. Self-presentation 2.0: narcissism and self-esteem on Facebook. Cyberpsychology, Behavior, and Social Networking. 2010;13(4): 357–64. doi: 10.1089/cyber.2009.0257 . 20712493

[35] Eksi F. Examination of Narcissistic Personality Traits' Predicting Level of Internet Addiction and Cyber Bullying through Path Analysis. Educational Sciences: Theory and Practice. 2012;12(3): 1694–706.

[36] Malik S, Khan M. Impact of facebook addiction on narcissistic behavior and self-esteem among students. Journal of the Pakistan Medical Association. 2015;65(3): 260–3. 25933557

[37] Ryan T, Xenos S. Who uses Facebook? An investigation into the relationship between the Big Five, shyness, narcissism, loneliness, and Facebook usage. Computers in Human Behavior. 2011;27(5): 1658–64. doi: 10.1016/j.chb.2011.02.004

[38] McCain JL, Campbell WK. Narcissism and Social Media Use: A Meta-Analytic Review. Psychology of Popular Media Culture. 2018;7(3): 308–27. doi: 10.1037/ppm0000137

[39] Brailovskaia J, Bierhoff H-W. Cross-cultural narcissism on Facebook: Relationship between self-presentation, social interaction and the open and covert narcissism on a social networking site in Germany and Russia. Computers in Human Behavior. 2016;55: 251–7. doi: 10.1016/j.chb.2015.09.018

[40] Buffardi LE, Campbell WK. Narcissism and social networking Web sites. Personality and Social Psychology Bulletin. 2008;34(10): 1303–14. doi: 10.1177/0146167208320061 . 18599659

[41] Carpenter CJ. Narcissism on Facebook: Self-promotional and anti-social behavior. Personality and Individual Differences. 2012;52(4): 482–6. doi: 10.1016/j.paid.2011.11.011

[42] Ong EY, Ang RP, Ho JC, Lim JC, Goh DH, Lee CS, et al. Narcissism, extraversion and adolescents’ self-presentation on Facebook. Personality and Individual Differences. 2011;50(2): 180–5. doi: 10.1016/j.paid.2010.09.022

[43] Ozimek P, Bierhoff H-W, Hanke S. Do vulnerable narcissists profit more from Facebook use than grandiose narcissists? An examination of narcissistic Facebook use in the light of self-regulation and social comparison theory. Personality and Individual Differences. 2018;124: 168–77. doi: 10.1016/j.paid.2017.12.016

[44] Casale S, Fioravanti G. Why narcissists are at risk for developing Facebook addiction: The need to be admired and the need to belong. Addictive Behaviors. 2018;76: 312–8. doi: 10.1016/j.addbeh.2017.08.038 28889060

[45] Kaur P, Dhir A, Chen S, Rajala R. Flow in context: Development and validation of the flow experience instrument for social networking. Computers in Human Behavior. 2016;59: 358–67. doi: 10.1016/j.chb.2016.02.039

[46] Mauri M, Cipresso P, Balgera A, Villamira M, Riva G. Why is Facebook so successful? Psychophysiological measures describe a core flow state while using Facebook. Cyberpsychology, Behavior, and Social Networking. 2011;14(12): 723–31. doi: 10.1089/cyber.2010.0377 21879884

[47] Csikszentmihalyi M. Flow: The psychology of optimal performance. New York, NY: Cambridge UniversityPress; 1990.

[48] Brailovskaia J, Rohmann E, Bierhoff H-W, Margraf J. The brave blue world: Facebook Flow and Facebook Addiction Disorder (FAD). PLoS ONE. 2018;13(7): e0201484. doi: 10.1371/journal.pone.0201484 30048544 PMC6062136

[49] Brailovskaia J, Teichert T. “I like it” and “I need it”: Relationship between implicit associations, flow, and addictive social media use. Computers in Human Behavior. 2020;13: 106509. doi: 10.1016/j.chb.2020.106509Get

[50] Brailovskaia J, Schillack H, Margraf J. Facebook Addiction Disorder (FAD) in Germany. Cyberpsychology, Behavior, and Social Networking. 2018;21(7): 450–6. doi: 10.1089/cyber.2018.0140 29995531

[51] Andreassen CS, Torsheim T, Brunborg GS, Pallesen S. Development of a Facebook addiction scale. Psychological Reports. 2012;110(2): 501–17. doi: 10.2466/02.09.18.PR0.110.2.501-517 22662404

[52] Andreassen CS, Griffiths MD, Gjertsen SR, Krossbakken E, Kvam S, Pallesen S. The relationships between behavioral addictions and the five-factor model of personality. Journal of Behavioral Addictions. 2013;2(2): 90–9. doi: 10.1556/JBA.2.2013.003 26165928

[53] American Psychiatric Association. Diagnostic and Statistical Manual of Mental Disorders (5th ed.). Washington, DC: American Psychiatric Association; 2013.

[54] World Health Organization. International classification of diseases for mortality and morbidity statistics (11th Revision) 2018. Available from: https://icd.who.int/browse11/l-m/en.

[55] Brailovskaia J, Teismann T, Margraf J. Physical activity mediates the association between daily stress and Facebook Addiction Disorder (FAD)–a longitudinal approach among German students. Computers in Human Behavior. 2018;86: 199–204. doi: 10.1016/j.chb.2018.04.045

[56] Brailovskaia J, Ströse F, Schillack H, Margraf J. Less Facebook use–More well-being and a healthier lifestyle? An experimental intervention study. Computers in Human Behavior. 2020;108: 106332. doi: 10.1016/j.chb.2020.106332

[57] Marino C, Gini G, Vieno A, Spada MM. A comprehensive meta-analysis on Problematic Facebook Use. Computers in Human Behavior. 2018;83: 262–77. doi: 10.1016/j.jad.2017.10.007 29024900

[58] Billieux J, Schimmenti A, Khazaal Y, Maurage P, Heeren A. Are we overpathologizing everyday life? A tenable blueprint for behavioral addiction research. Journal of Behavioral Addictions. 2015;4(3): 119–23. doi: 10.1556/2006.4.2015.009 26014667 PMC4627665

[59] Carbonell X, Panova T. A critical consideration of social networking sites’ addiction potential. Addiction Research & Theory. 2017;25(1): 48–57.

[60] Brailovskaia J, Rohmann E, Bierhoff H-W, Margraf J, Köllner V. Relationships between addictive Facebook use, depressiveness, insomnia, and positive mental health in an inpatient sample: A German longitudinal study. Journal of Behavioral Addictions. 2019;8(4): 703–13. doi: 10.1556/2006.8.2019.63 31830811 PMC7044577

[61] Brailovskaia J, Teismann T, Margraf J. Positive mental health mediates the relationship between Facebook Addiction Disorder and suicide-related outcomes: A longitudinal approach. Cyberpsychology, Behavior, and Social Networking. 2020. doi: 10.1089/cyber.2019.0563 32216638

[62] Brailovskaia J, Schillack H, Margraf J. Tell me why are you using social media (SM)! Relationship between reasons for use of SM, SM flow, daily stress, depression, anxiety, and addictive SM use–An exploratory investigation of young adults in Germany. Computers in Human Behavior. 2020;113: 106511. doi: 10.1016/j.chb.2020.106511

[63] Atroszko PA, Balcerowska JM, Bereznowski P, Biernatowska A, Pallesen S, Andreassen CS. Facebook addiction among Polish undergraduate students: Validity of measurement and relationship with personality and well-being. Computers in Human Behavior. 2018;85: 329–38. doi: 10.1016/j.chb.2018.04.001

[64] Koc M, Gulyagci S. Facebook addiction among Turkish college students: The role of psychological health, demographic, and usage characteristics. Cyberpsychology, Behavior, and Social Networking. 2013;16(4): 279–84. doi: 10.1089/cyber.2012.0249 23286695

[65] Brailovskaia J, Margraf J, Köllner V. Addicted to Facebook? Relationship between Facebook Addiction Disorder, duration of Facebook use and narcissism in an inpatient sample. Psychiatry Research. 2019;273: 52–7. doi: 10.1016/j.psychres.2019.01.016 30639564

[66] Błachnio A, Przepiórka A. Facebook intrusion, fear of missing out, narcissism, and life satisfaction: A cross-sectional study. Psychiatry Research. 2018;259: 514–9. doi: 10.1016/j.psychres.2017.11.012 29154204

[67] Mayr S, Erdfelder E, Buchner A, Faul F. A short tutorial of GPower. Tutorials in Quantitative Methods for Psychology. 2007;3(2): 51–9. doi: 10.20982/tqmp.03.2.p051

[68] Hayes AF. Introduction to mediation, moderation, and conditional process analysis. London: Guilford Press; 2013.

[69] Urban D, Mayerl J. Regressionsanalyse: Theorie, Technik und Anwendung (2. Aufl.). Wiesbaden: VS Verlag für Sozialwissenschaften; 2006.

[70] Andreassen CS, Pallesen S, Griffiths MD. The relationship between addictive use of social media, narcissism, and self-esteem: Findings from a large national survey. Addictive Behaviors. 2017;64: 287–93. doi: 10.1016/j.addbeh.2016.03.006 27072491

[71] Rose P. The happy and unhappy faces of narcissism. Personality and Individual Differences. 2002;33(3): 379–91.

[72] Xie W, Karan K. Predicting Facebook addiction and state anxiety without Facebook by gender, trait anxiety, Facebook intensity, and different Facebook activities. Journal of Behavioral Addictions. 2019;8(1): 79–87. doi: 10.1556/2006.8.2019.09 30880400 PMC7044595

[73] Wolniewicz CA, Tiamiyu MF, Weeks JW, Elhai JD. Problematic smartphone use and relations with negative affect, fear of missing out, and fear of negative and positive evaluation. Psychiatry Research. 2018;262: 618–23. doi: 10.1016/j.psychres.2017.09.058 28982630

[74] Elhai JD, Yang H, Fang J, Bai X, Hall BJ. Depression and anxiety symptoms are related to problematic smartphone use severtity in Chinese young adults: Fear of missing out as a mediator. Addictive Behaviors. 2020;101: 1–7. doi: 10.1016/j.addbeh.2019.04.020 31030950

[75] Elhai JD, Levine JC, Alghraibeh AM, Alafnan AA, Aldraiweesh AA, Hall BJ. Fear of missing out: Testing relationships with negative affectivity, online social engagement, and problematic smartphone use. Computers in Human Behavior. 2018;89: 289–98. doi: 10.1016/j.chb.2018.08.020

[76] Clayton RB, Osborne RE, Miller BK, Oberle CD. Loneliness, anxiousness, and substance use as predictors of Facebook use. Computers in Human Behavior. 2013;29(3): 687–93. doi: 10.1016/j.chb.2012.12.002

[77] Miller JD, Hoffmann B, Gaughan ET, Gentile B, Maples J, Campbell WK. Grandiose and vulnerable narcissism: A nomological network analyses. Journal of Personality. 2011;79: 1013–42. doi: 10.1111/j.1467-6494.2010.00711.x 21204843

[78] Kim EJ, Namkoong K, Ku T, Kim SJ. The relationship between online game addiction and aggression, self-control and narcissistic personality traits. European Psychiatry. 2008;23(3): 212–8. doi: 10.1016/j.eurpsy.2007.10.010 18166402

[79] Miller JD, Gentile B, Carter NT, Crowe M, Hoffman BJ, Campbell WK. A Comparison of the Nomological Networks Associated With Forced-Choice and Likert Formats of the Narcissistic Personality Inventory. Journal of Personality Assessment. 2018;100(3): 259–67. doi: 10.1080/00223891.2017.1310731 28436690

[80] Błachnio A, Przepiorka A, Rudnicka P. Narcissism and self-esteem as predictors of dimensions of Facebook use. Personality and Individual Differences. 2016;90: 296–301. doi: 10.1016/j.paid.2015.11.018

[81] Chen W, Lee K-H. Sharing, liking, commenting, and distressed? The pathway between Facebook interaction and psychological distress. Cyberpsychology, Behavior, and Social Networking. 2013;16(10): 728–34. doi: 10.1089/cyber.2012.0272 23745614

[82] Kraemer HC, Kazdin AE, Offord DR, Kessler RC, Jensen PS, Kupfer DJ. Coming to terms with the terms of risk. Archives of General Psychiatry. 1997;54(4): 337–43. doi: 10.1001/archpsyc.1997.01830160065009 9107150

[83] Campbell WK. When you love a man who loves himself. Naperville, IL: Sourcebooks; 2005.

[84] Green A, MacLean R, Charles K. Unmasking gender differences in narcissism within intimate partner violence. Personality and Individual Differences. 2020;167: 110247. doi: 10.1016/j.paid.2020.110247

[85] Ryan KM, Weikel K, Sprechini G. Gender differences in narcissism and courtship violence in dating couples. Sex Roles. 2008;58(11–12): 802–13. doi: 10.1007/s11199-008-9403-9

[86] Gentile B, Miller JD, Hoffman BJ, Reidy DE, Zeichner A, Campbell WK. A test of two brief measures of grandiose narcissism: the narcissistic personality inventory-13 and the narcissistic personality inventory-16. Psychological Assessment. 2013;25(4): 1120–36. doi: 10.1037/a0033192 . 23815119

[87] Ackerman RA, Donnellan MB, Roberts BW, Fraley RC. The effect of response format on the psychometric properties of the Narcissistic Personality Inventory: Consequences for item meaning and factor structure. Assessment. 2016;23(2): 203–20. doi: 10.1177/1073191114568113 25616401

[88] Raskin R, Terry H. A principal-components analysis of the Narcissistic Personality Inventory and further evidence of its construct validity. Journal of Personality and Social Psychology. 1988;54(5): 890–902. doi: 10.1037//0022-3514.54.5.890 3379585

[89] Back MD, Küfner AC, Dufner M, Gerlach TM, Rauthmann JF, Denissen JJ. Narcissistic admiration and rivalry: Disentangling the bright and dark sides of narcissism. Journal of Personality and Social Psychology. 2013;105(6): 1013. 24128186 10.1037/a0034431

[90] Campbell WK, Bonacci AM, Shelton J, Exline JJ, Bushman BJ. Psychological entitlement: Interpersonal consequences and validation of a self-report measure. Journal of Personality Assessment. 2004;83(1): 29–45. doi: 10.1207/s15327752jpa8301_04 15271594

[91] Sauley KS, Bedeian AG. Equity sensitivity: Construction of a measure and examination of its psychometric properties. Journal of Management. 2000;26(5): 885–910. doi: 10.1177/014920630002600507

[92] Miller BK. Confirmatory factor analysis of the equity preference questionnaire. Journal of Managerial Psychology. 2009;24(4): 328–47. doi: 10.1108/02683940910952714

[93] Miller BK, Gallagher DG. Examining trait entitlement using the self-other knowledge asymmetry model. Personality and Individual Differences. 2016;92: 113–7. doi: 10.1016/j.paid.2015.12.030

[94] Kircaburun K, Griffiths MD. Instagram addiction and the Big Five of personality: The mediating role of self-liking. Journal of Behavioral Addictions. 2018;7(1): 158–70. doi: 10.1556/2006.7.2018.15 29461086 PMC6035031

[95] Griffiths MD, Kuss DJ, Demetrovics Z. Social Networking Addiction: An Overview of Preliminary Findings. In: Rosenberg KP, Feder LC, editors. Behavioral Addictions. San Diego: Academic Press; 2014. p. 119–41

